# Origins of the Vertebrate Erythro/Megakaryocytic System

**DOI:** 10.1155/2015/632171

**Published:** 2015-10-18

**Authors:** Ondrej Svoboda, Petr Bartunek

**Affiliations:** Department of Cell Differentiation, Institute of Molecular Genetics AS CR v.v.i., 142 20 Prague 4, Czech Republic

## Abstract

Vertebrate erythrocytes and thrombocytes arise from the common bipotent thrombocytic-erythroid progenitors (TEPs). Even though nonmammalian erythrocytes and thrombocytes are phenotypically very similar to each other, mammalian species have developed some key evolutionary improvements in the process of erythroid and thrombocytic differentiation, such as erythroid enucleation, megakaryocyte endoreduplication, and platelet formation. This brings up a few questions that we try to address in this review. Specifically, we describe the ontology of erythro-thrombopoiesis during adult hematopoiesis with focus on the phylogenetic origin of mammalian erythrocytes and thrombocytes (also termed platelets). Although the evolutionary relationship between mammalian and nonmammalian erythroid cells is clear, the appearance of mammalian megakaryocytes is less so. Here, we discuss recent data indicating that nonmammalian thrombocytes and megakaryocytes are homologs. Finally, we hypothesize that erythroid and thrombocytic differentiation evolved from a single ancestral lineage, which would explain the striking similarities between these cells.

## 1. Introduction

**Figure 1:**
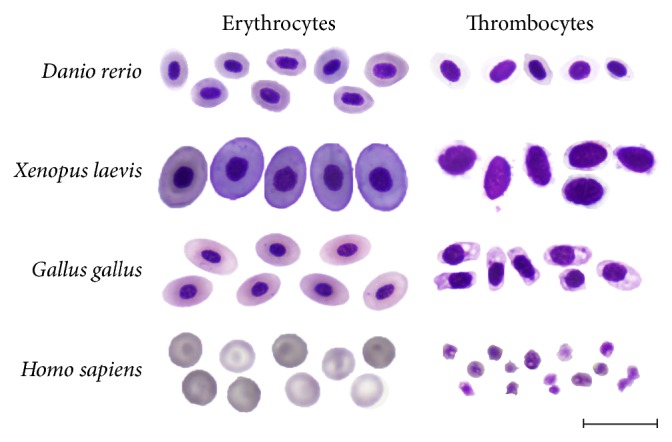
A comparative view of erythrocytes and thrombocytes from zebrafish (*Danio rerio*), xenopus (*Xenopus laevis*), chicken (*Gallus gallus*), or human (*Homo sapiens*) peripheral blood. Cells were smeared on glass slides and stained with May-Grünwald Giemsa. Photomicrographs were taken at 1000x magnification. Scale bar is 20 *μ*m.

Hematopoiesis is mediated by self-renewal and differentiation of hematopoietic stem cells (HSCs) and their progenies, which is tightly controlled through a complex array of extrinsic and intrinsic factors [1, 2]. Dysregulation of some of these pathways can lead to distinct hematopoietic disorders, such as anemia, thrombocytopenia, and myelogenous leukemia, predominantly caused by defects in the erythroid-megakaryocytic compartment [3, 4]. It is well accepted that mammalian megakaryocytes and erythrocytes are generated from common bipotent megakaryocyte-erythrocyte progenitors (MEPs) [5]. In mammals, megakaryocytes are formed by endoreduplication of megakaryoblasts to generate polyploid cells. Once the ploidy state of 8–64N is reached, megakaryocytes produce thrombocytes (in mammals also referred to as platelets) [6]. The key mediator of this process is thrombopoietin (TPO) [7, 8]. Red blood cells (RBCs) do likewise develop from MEPs through several stages of committed progenitors, termed burst-forming units-erythroid (BFU-E), colony-forming units-erythroid (CFU-E), and erythroblasts. The most prominent factors regulating erythropoiesis* in vivo* and* ex vivo* are erythropoietin (EPO) and stem cell factor (SCF, or KIT ligand, KITL) [9, 10]. Notably, mammalian erythroblasts undergo chromatin condensation and nucleus extrusion, giving rise to enucleated mature erythrocytes [11, 12]. In contrast, nonmammalian vertebrates possess nucleated oval-shaped diploid thrombocytes [13, 14] and RBCs [15] (Figure 1). Similarly to mammals, both of these lineages have been demonstrated to arise from bipotent progenitors, termed thrombocyte-erythrocyte progenitors (TEPs), cells equivalent to mammalian MEPs [16, 17].

The present review aims to summarize the ontology and phylogeny of erythro-thrombocytic differentiation in vertebrates. Here, we highlight the relationship between mammalian and nonmammalian erythroid and thrombocytic cells. Moreover, despite the morphological and functional differences between erythroid and thrombocytic cell lineages, we provide a model underlining the common evolutionary origin of these two cell lineages from a single ancestral precursor.

## 2. Ontogeny of Thrombocytes and Erythrocytes

### 2.1. Models of Adult Hematopoiesis

**Figure 2:**
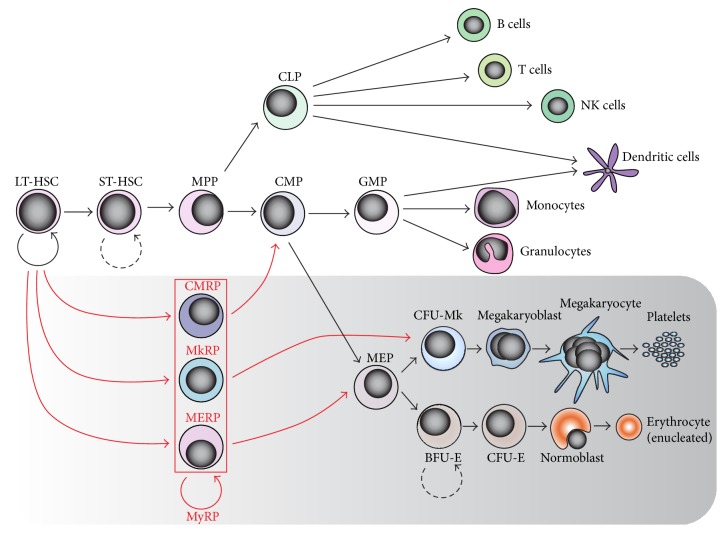
Models of mammalian adult hematopoiesis with respect to the megakaryocytic-erythroid compartment (grey box). Hierarchical [20, 21] (black arrows) and myeloid bypass [25] (red arrowheads) models of hematopoiesis are shown. According to the conventional hierarchical model of hematopoiesis, the bipotent megakaryocyte-erythroid progenitors (MEPs) are able to give rise to megakaryocytes and erythrocytes. The alternative myeloid bypass model predicts the existence of various myeloid repopulating progenitors (MyRPs) as a subset of long-term hematopoietic stem cells (LT-HSCs), such as common myeloid repopulating progenitors (CMRPs), megakaryocyte repopulating progenitors (MkRPs), and megakaryocyte-erythroid repopulating progenitors (MERPs). These progenitors are capable of long-term repopulation and differentiation into the particular cell lineages. ST-HSC: short-term HSC; MPP: multipotent progenitor cell; CLP: common lymphoid progenitor; GMP: granulocyte/monocyte progenitor; CFU-Mk: colony-forming unit-megakaryocyte; BFU-E: burst-forming units-erythroid; CFU-E: colony-forming units-erythroid.

Both* in vivo* and* ex vivo*, all terminally differentiated blood cells in adult organisms arise from long-term HSCs (LT-HSC) [18, 19] that have unlimited self-renewal capacity. Direct downstream progenies of HSCs, termed short-term HSCs (ST-HSCs) and multipotent progenitor cells (MPPs), are progressively losing their self-renewal capability upon commitment. According to the most prevalent classical hierarchical model of hematopoiesis (Figure 2) [20, 21], the MPPs further give rise to common lymphoid progenitors (CLPs) and common myeloid progenitors (CMPs). CLPs are responsible for production of lymphoid cells, whereas CMPs differentiate into granulocyte-monocyte progenitors (GMPs) and bipotent MEPs/TEPs that are responsible for generation of thrombocytes and erythrocytes [6, 16, 17], the most abundant and specialized cell types in the adult organism.

Although the hierarchical model of hematopoiesis has been generally accepted over years, recent development of state-of-the-art technologies led to discoveries of alternative hematopoietic pathways that are either bias or bypass certain multipotent progenitors. This includes the myeloid/lymphoid biased model [22], revised model for adult hematopoiesis [23], myeloid based model [24], or myeloid bypass model [25]. Some of these models are in accordance with the classical hierarchical model and provide alternative pathways for development of more specialized cell types at a much higher hierarchical level than previously realized. Although detailed examination of these pathways goes beyond the scope of this review, we would like to highlight those alternative models that refer to production of erythroid and megakaryocytic cells (Figure 2).

First evidence suggesting a direct pathway leading from HSCs to MEPs was based on the identification of a subset of HSCs, marked by Lineage^−^Sca1^+^c-Kit^high^ (LSK) and Flt3^−^ antibodies, which may have given rise directly to MEPs [22]. This is in agreement with recent findings, further proving that the LSK CD150^+^CD48^−^CD34^−^ subset of HSCs is capable of short-term and long-term platelet reconstitution as well as reconstitution of other erythro-myeloid, but not lymphoid, cell lineages [26]. Extensive single-cell transplantation experiments revealed the presence of long-term megakaryocyte repopulating progenitors (MkRPs), megakaryocyte-erythroid repopulating progenitors (MERPs), and common myeloid repopulating progenitors (CMRPs) within the CD150^+/−^CD41^+/−^CD34^−^LSK cells (Figure 2) [25]. While CMRPs were shown to be generally present within the CD34^−^LSK fraction of cells, MkRPs and MERPs seem to be present only within the CD150^+^CD41^−^CD34^−^LSK or CD150^−^CD41^+^CD34^−^LSK fraction of cells. As a follow-up, the intermediate pathway bridging MkRP and megakaryocytes was identified, and fully restricted unipotent megakaryocyte progenitors CD41^+^CD42b^+^LSK were characterized recently [27].

Importantly, the experimental data suggesting alternative erythroid and megakaryocytic pathways are solely based on experiments performed in mammalian hematopoietic models and there is a lack of evidence of their existence in nonmammalian vertebrate species. We can only speculate whether these pathways evolved in mammals only or whether they are evolutionarily conserved. One may presume that these mechanisms might play an important physiological role in the steady-state and emergency hematopoiesis.

### 2.2. Extrinsic Factors Involved in Erythro-Thrombopoiesis

**Figure 3:**
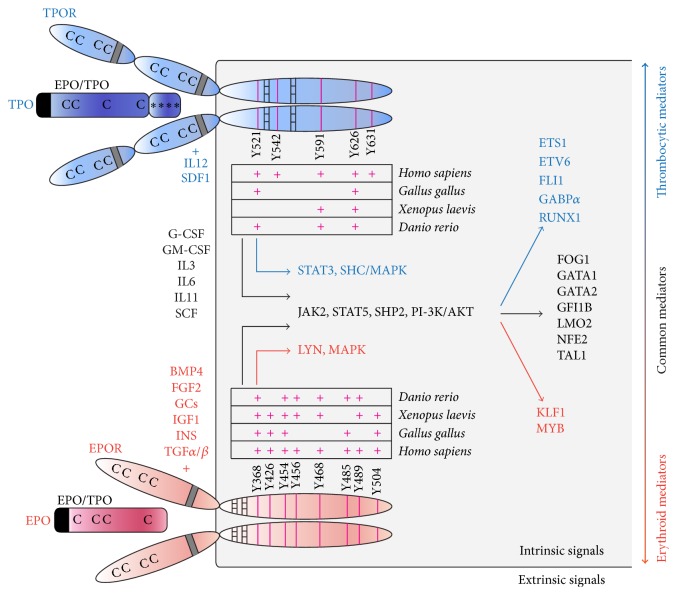
Composite summary of the most prominent factors and signals involved in the regulation of erythro-thrombopoiesis. Erythroid signals are in red, thrombocytic signals are in blue, and signals involved in both differentiation pathways are depicted in black. Human TPO ligand and TPOR are shown in blue; human EPO and EPOR are shown in red. Tyrosine residues (Y, pink lines) in TPOR/EPOR intracellular domains important for receptor signaling are shown. Some of them are highly conserved throughout the vertebrate species as demonstrated in the table. Cytokines' signal peptides are in black and EPO/TPO domains are shown. Receptors' WSXSW motifs are in grey and squared boxes represent Box1/Box2. Cs represent conserved Cys residues; *∗*s represent glycosylation sites.

Erythro-thrombocytic differentiation has been shown to be regulated by multiple cytokines (Figure 3), many of which have broad effects on all hematopoietic lineages. The most important factors regulating erythropoiesis are EPO and SCF [9, 10]. EPO interacts with its cognate receptor, EPOR, and promotes erythroid progenitor self-renewal, survival, and differentiation, while SCF mediates proliferation of these progenitors. Other important factors that control erythropoiesis include fibroblast growth factor 2 (FGF2) [28], insulin (INS), insulin-like growth factor 1 (IGF1) [29], transforming growth factor *α* (TGF*α*) and TGF*β* family members (TGF*β*, bone morphogenetic protein 4, BMP4) [30–34], and glucocorticoids (GCs, such as dexamethasone, Dex) [35]. These factors could either promote erythroid progenitor self-renewal or take part in their differentiation, depending on the cooperating signals. Further cytokines that act synergistically with the lineage-restricted factors and that could instrument both erythroid and thrombocytic differentiation pathways are interleukin 3 (IL3), IL6, IL11, granulocyte-colony stimulating factor (G-CSF), granulocyte-macrophage CSF (GM-CSF) [36, 37], and the previously mentioned SCF [10, 38]. These factors act as early modulators of upstream progenitors in erythroid and thrombocytic differentiation, driving their self-renewal, or promote megakaryocytic maturation. Other cytokines involved in thrombocytic differentiation, megakaryocytic maturation, or platelet biogenesis include, besides TPO, also IL12 and SDF1 [36]. TPO interacts with its cognate receptor, TPOR (c-MPL) [39, 40]. Its signalization seems to be strongly required for thrombopoiesis, since mice lacking c-MPL signaling are highly thrombocytopenic [41].

It is interesting that EPO and TPO signaling share many common features. Both ligands belong to the four-helix bundle cytokine family and share a highly conserved amino-terminal EPO/TPO domain [42]. In mammals, TPO's C-terminal portion encodes a highly glycosylated domain [43, 44] that is missing in nonmammalian vertebrates [16, 17] and its role in mammals is to regulate the half-life of TPO in the circulation [41]. EPO and TPO share four conserved cysteine (Cys) residues that form disulfide bonds [16, 17, 45] responsible for keeping the ligand's tertiary structure. Importantly, EPOR and TPOR are also reminiscent of each other. Both receptors belong to the family of type I cytokine receptors [46, 47]. The extracellular domains of these receptors [48] are characterized by the presence of four conserved Cys residues and a tryptophan-serine-x-serine-tryptophan (WSXSW) motif, involved in ligand binding and receptor signaling. Class I receptors possess one transmembrane domain, and their intracellular region consists of two conserved domains, Box1/Box2, involved in mediating downstream signals. Other important features of these receptors are intracellular tyrosine residues, many of which are conserved throughout the vertebrate species (Figure 3). The only structural difference between EPOR and TPOR is that the extracellular domain has been duplicated in TPOR, having eight conserved Cys residues and two WSXSW motifs [46]. The activation of EPOR as well as TPOR occurs through the receptor homodimerization upon ligand binding, which in turn triggers similar downstream signaling pathways. These similarities between EPO/TPO ligands and their receptors suggest that they might have evolved from a single ligand/receptor by a duplication event during evolution.

### 2.3. Intrinsic Factors Involved in Erythro-Thrombopoiesis

The intracellular signaling pathways mediated by EPOR and TPOR overlap to a large extent (Figure 3). Neither EPOR nor TPOR have an intrinsic enzymatic activity and their signaling is primarily dependent on associated Janus kinase 2 (JAK2) [49, 50]. Particularly, receptor homodimerization leads to autophosphorylation of JAK2 that is bound to Box1/2 and that in turn phosphorylates the receptor itself as well as other signaling molecules. Both EPOR and TPOR stimulate JAK2-mediated phosphorylation of STAT5 (signal transducers and activators of transcription), activate the phosphatidylinositol 3-kinase (PI-3K)/AKT pathway, and promote mitogen-activated protein kinase (MAPK) signaling [49, 50]. This is achieved by recruitment of GRB2 either directly or indirectly via the adaptor molecule SHC, while GRB2 further activates SOS, RAF, and MEK proteins, finally triggering MAPK activation [51, 52]. In contrast to EPOR, TPOR is a much more potent activator of the MAPK pathway and STAT3 signaling [53]. Conversely, it has been shown that EPOR interacts with LYN kinase, which can bind to JAK2 and affects STAT5 [54]. EPOR and TPOR signaling is limited by a negative feedback loop employing SHP1 and SHIP phosphatases and suppressors of cytokine signaling (SOCS1, SOCS3) [49, 55, 56].

The described signaling networks work either in concert or antagonistically to drive specification of erythroid and thrombocytic cell lineages. This is mainly governed by the balanced activity of transcription factors binding to GATA or ETS motifs and others [57], including GATA binding factors, GATA1, GATA2; ETS factors, ETS1, ETS Variant 6 (ETV6/TEL), friend leukemia virus integration 1 (FLI1), GA-binding protein transcription factor (GABP*α*); and other factors, runt-related transcription factor 1 (RUNX1/AML1), c-MYB (MYB), friend of GATA1 (FOG1), growth factor independent 1B (GFI1B), nuclear factor-erythroid2 complex (NFE2, NFE2, and MAFK subunits), LIM domain only 2 (LMO2), T-cell acute lymphocytic leukemia 1 (TAL1/SCL), and Krüppel-like factor (KLF1) [3, 57–59]. It is the interaction and crosstalk between these transcription factors that makes the system complex. A number of these transcription factors, such as FOG1, GATA1/2, GFI1B, LMO2, NFE2, and TAL1, are critical for both erythroid and thrombocytic development, whereas others are rather dedicated to unilineage differentiation, such as the erythroid EKLF and MYB or the thrombocytic ETS1, ETV6, FLI1, GABP*α*, and RUNX1 (Figure 3). From this overview, it is more than apparent that erythroid and thrombocytic signaling share many common features, further suggesting a common evolutionary origin of EPO/TPO signaling pathways.

## 3. Phylogeny of Erythrocytes and Thrombocytes in Vertebrates

Mammalian and nonmammalian erythro-thrombocytic cells appear to be phenotypically very different as a result of divergent evolution. It has been shown that mammals and birds split off from their lizard-like ancestors 310 million years ago [60]. Since that time, certain aspects of erythroid and thrombocytic differentiation have changed; adult mammalian RBCs possess the unique feature of being enucleated, and mammalian thrombocytes are not individual cells but fragments of megakaryocytes. These adaptations likely enhanced the biological performance of the corresponding cells, which could be demonstrated on a few examples. Enucleated erythrocytes are more flexible and the lack of the nucleus creates more intracellular space for hemoglobin. This provides a typical biconcave shape, increasing the surface area for an efficient oxygen exchange [61]. Mammalian platelets are generated in very high numbers (thousands of platelets per one megakaryocyte), as compared to nonmammalian thrombocytes, and are much smaller and more flexible. These features ensure their efficient spreading and increased resistance to fluid shear forces [62]. Both of these improvements in erythrocytes and thrombocytes allowed development of thinner capillaries in mammals, preventing their possible blockage [61, 62]. It is likely that these enhancements provided a survival advantage to early mammalian species.

**Figure 4:**
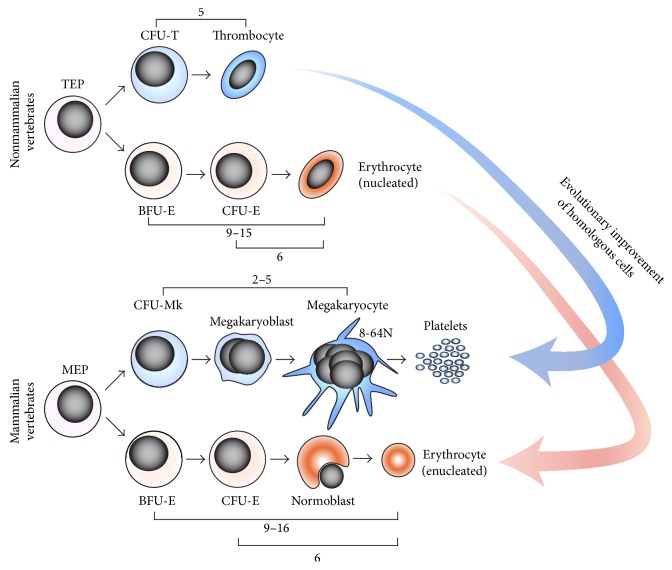
Nonmammalian and mammalian model of erythropoiesis and thrombopoiesis. According to the integrated model of hematopoiesis, mammalian erythrocytes and megakaryocytes have likely evolved from their nonmammalian erythroid and thrombocytic homologs as an evolutionary improvement. Nonmammalian erythrocytes and thrombocytes are phenotypically similar (nucleated, diploid oval-shaped cells), whereas mammalian megakaryocytes and erythrocytes are very different from each other. Numbers indicate the proliferation potential of particular progenitors. TEP: thrombocyte-erythroid progenitor; CFU-T: colony-forming unit-thrombocyte. Modified from Bartunek et al. [16] and Svoboda et al. [17].

However, these enhancements also bring up the question of the evolutionary origin of these cells. Hypothetically, mammalian erythrocytes and megakaryocytes could have evolved* de novo*, functioning as analogs of nonmammalian erythrocytes and thrombocytes [17]. Conversely, they might have evolved as a possible improvement from ancestral erythro-thrombocytic cells, as previously discussed, indicating that mammalian and nonmammalian erythrocytes and thrombocytes are homologs. Indeed, the following lines of evidence suggest that the latter hypothesis may be more probable (Figure 4).

First, the initial commitment of both lineages requires involvement of similar signaling pathways and transcription factors throughout the vertebrate phylum. The most prominent factors required for erythroid differentiation that were found to be functionally conserved from fish to man are FOG1, GATA1, GATA2, KLF1 (zebrafish ortholog Klf4), LMO2, MYB, NFE2, TAL1, and others [17, 63–66]. Similarly, the list of conserved factors that are involved in vertebrate thrombopoiesis includes ETS1, FLI1, FOG1, GATA1, GATA2, NFE2, RUNX1, TAL1, and others [17, 64, 67–69]. Second, multiple zebrafish mutant lines or knockdowns have been generated that recapitulate common human disorders, such as various types of anemia, protoporphyria, or thrombocytopenia [64, 70, 71]. Many of these mutants and knockdowns are affected in the same loci that are relevant to human diseases, which further highlights the similar mechanisms underpinning these processes. Third, the processes involved in hemostasis are highly conserved among mammalian and nonmammalian vertebrates; platelets and thrombocytes are activated by the same stimuli, and blood clotting takes place in an almost identical way [62, 71].

Finally, the last piece of evidence favoring the hypothesis that megakaryocytes likely evolved as a thrombocytic improvement is based on characterization of the relationships between zebrafish hematopoietic progenitors and on mapping their proliferation kinetics. Multiple studies indicate that mammalian BFU-E progenitors are capable of 9 to 16 cell divisions during their maturation, depending on the presence of cooperating factors [72]. The CFU-E progenitors are capable of at most 6 cell divisions [72] and megakaryocytes endoreduplicate approximately 2 to 5 times [73] to form 8–64N cells. In line with this observation is the study indicating that the number of cell divisions during zebrafish erythroid and thrombocytic terminal differentiation is closely matched to mammalian species [17]: the zebrafish BFU-E progenitors are capable of 9 to 15 cell divisions, depending on cooperative signals, the CFU-E progenitors can undergo 6 cell divisions, and thrombocytes can undergo 5 cell divisions during their terminal differentiation.

Taken together, these data led to the establishment of the “integrated model of hematopoiesis” [17] (Figure 4), proposing that despite striking phenotypic differences between mammalian megakaryocytes and nonmammalian thrombocytes, there is a clear link between mammalian and nonmammalian erythroid and thrombocytic cells in terms of their molecular control and their proliferation potential. This model further suggests that mammalian erythrocytes and megakaryocytes have evolved from nonmammalian erythrocytes and thrombocytes as their possible improvements, which implies their homologous relationship.

## 4. Origin of Erythrocytes and Thrombocytes in Vertebrates

**Figure 5:**
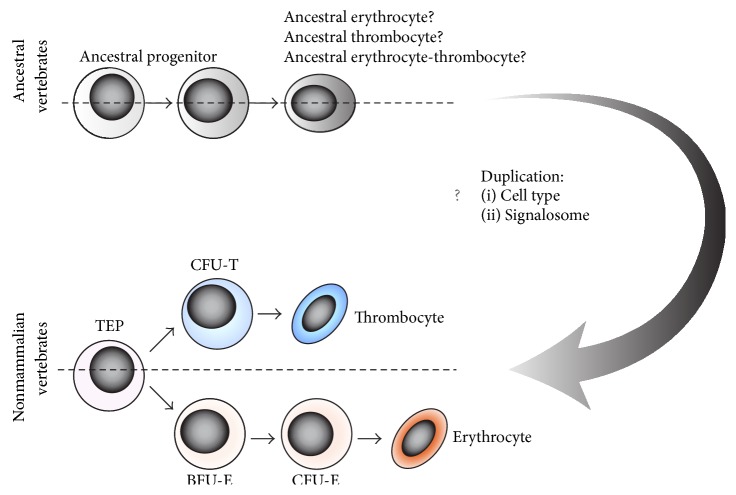
The common ancestral erythro-thrombocytic model predicts the existence of unilineage differentiation in ancestral vertebrates, leading to ancient erythroid or thrombocytic cells or erythro-thrombocytic cells with dual function. Hypothetical duplication of cell types and their signalosomes led to the origin of erythrocytes with EPO signaling and to the origin of thrombocytes together with TPO signaling.

Up to now, we have discussed the evolutionary development of mammalian erythrocytes and megakaryocytes from nonmammalian homologous cells. However, in this chapter we would like to focus on the hypothetical origin of erythroid and thrombocytic differentiation programs from ancestral vertebrates. According to the generally well-accepted evolutionary hypothesis, the invertebrate and vertebrate species bifurcated approximately 520–550 million years ago [60]. This resulted in enormous divergence of these species and led to* de novo* parallel formation of various analogous features. Even though many invertebrate animals possess both erythrocyte-like and thrombocyte-like analogous cells, commonly referred to as amebocytes, coelomocytes, hemocytes, or thrombocytoids, these cells are not considered to be the progenitors of vertebrate erythro-thrombocytic cells [74–77]. Therefore, erythrocytes and thrombocytes found in cyclostomates and fish are the first cells that have evolved to be particularly specialized in oxygen transport or hemostasis [13, 78–80]. Both cell lineages likely first appeared in direct fish ancestors and it is highly probable that both differentiation programs split from one ancestral differentiation program after its duplication (Figure 5). This view is supported both by similar cell characteristics (similar oval shape, condensed nuclei, and proliferation coupled to differentiation) and by similar or shared regulatory molecules, as previously discussed. This includes the structural and functional resemblance between EPO and TPO signalosomes, likely derived from a single ligand-receptor complex due to a duplication event. As discussed above, both EPO and TPO mediate substantially redundant signaling and activate similar signaling pathways and transcription factors. This has been well illustrated experimentally as TPO expanded erythroid progenitors [7, 81] and, strikingly, TPO in combination with SCF and IL11 was shown to substitute for EPO signaling in the erythroid progenitors derived from* Epor* deficient mice [82]. Conversely, EPO was shown to synergize with TPO to promote megakaryocyte colony growth and maturation [36, 83].

In summary, based on the integrated model of hematopoiesis we propose the “Common ancestral erythro-thrombocytic hypothesis.” This hypothesis predicts the existence of ancestral vertebrate organisms with unilineage differentiation, leading to ancestral erythrocytes/thrombocytes or erythro-thrombocytes with dual function. This unilineage differentiation program might have been further duplicated during the evolution of early vertebrates, giving rise to specialized erythroid and thrombocytic differentiation programs in conjunction with EPO/TPO signaling.

## 5. Conclusions

Erythroid and thrombocytic differentiation share many common features. Besides phenotypic similarities between erythrocytes and thrombocytes found in nonmammalian vertebrates, this includes the common progenitors of these cells (TEPs/MEPs), similarities between EPO and TPO signaling, and shared signaling mechanisms mediating the lineage commitment. These similarities are also present in mammalian species, while the basic molecular mechanisms driving erythro-thrombocytic lineage commitment seem to be evolutionarily highly conserved. The integrated model of hematopoiesis (Figure 5) suggests that mammalian megakaryocytes and erythrocytes likely evolved as an improvement of their ancestral counterparts (found in nonmammalian vertebrates) to increase their biological performance during oxygen transport and hemostasis. This indicates that mammalian and nonmammalian erythrocytes and thrombocytes did not evolve* de novo* but instead are homologous.

Finding the actual relationship between the mammalian and nonmammalian blood cells might have a major impact on hematopoietic research. Since the employment of mammalian model organisms brings only partial progress due to the interference with sophisticated mammalian megakaryocytic and erythroid enhancements, nonmammalian model organisms, such as chicken or zebrafish, could then be efficiently utilized to identify novel key regulators of cell fate determination.

In addition to this and based on the described similarities between erythroid and thrombocytic differentiation, we suggest that both cell lineages have evolved from a single ancestral differentiation program. This was likely mediated by the duplication of the ancestral cell type and its signalosome during the evolution of early vertebrates.
